# Working with suicidal mothers during the perinatal period: a reflexive thematic analysis study with mental health professionals

**DOI:** 10.1186/s12888-024-05537-1

**Published:** 2024-02-07

**Authors:** Holly E. Reid, Dawn Edge, Daniel Pratt, Anja Wittkowski

**Affiliations:** 1 Division of Psychology and Mental Health, School of Health Sciences, Faculty of Biology, Medicine and Health, University of Manchester, Zochonis Building, Brunswick Street, M13 9PL Manchester, UK; 2 Manchester Academic Health Sciences Centre, Manchester, UK; 3 Greater Manchester Mental Health NHS Foundation Trust, Manchester, UK

**Keywords:** Staff, Clinicians, Professionals, Perinatal, Suicide, Qualitative, Interviews

## Abstract

**Background:**

Suicide is the leading cause of death in mothers postpartum and one of the most common causes of death during pregnancy. Mental health professionals who work in perinatal services can offer insights into the factors they perceive as being linked to mothers’ suicidal ideation and behaviour, support offered to mothers and improvements to current practices. We aimed to explore the experiences and perceptions of perinatal mental health professionals who have worked with suicidal mothers during the perinatal period.

**Method:**

Semi-structured interviews were conducted face-to-face or via telephone with mental health professionals working in perinatal mental health inpatient or community services across England. Data were analysed using reflexive thematic analysis.

**Results:**

From the professionals’ (*n* = 15) accounts three main themes were developed from their interview data. The first, *factors linked to suicidal ideation and behaviour*, overarched two sub-themes: (1.1) *the mother’s context* and (1.2) *what the baby represents and what this means for the mother*. These sub-themes described factors that professionals assessed or deemed contributory in relation to suicidal ideation and behaviour when a mother was under their care. The second main theme, *communicating about and identifying suicidal ideation and behaviour*, which outlined how professionals enquired about, and perceived, different suicidal experiences, encapsulated two sub-themes: (2.1) *how to talk about suicide* and (2.2) *types of suicidal ideation and attempts.* The third main theme, *reducing suicidal ideation through changing how a mother views her baby and herself*, focused on how professionals supported mothers to reframe the ways in which they viewed their babies and in turn themselves to reduce suicidal ideation.

**Conclusion:**

Professionals highlighted many factors that should be considered when responding to a mother’s risk of suicide during the perinatal period, such as the support around her, whether the pregnancy was planned and what the baby represented for the mother. Professionals’ narratives stressed the importance of adopting a tailored approach to discussing suicidal experiences with mothers to encourage disclosure. Our findings also identified psychological factors that professionals perceived as being linked to suicidal outcomes for mothers, such as self-efficacy; these factors should be investigated further.

## Introduction

Mental disorders experienced during pregnancy and the first postpartum year (i.e. the perinatal period) are the most common complication of childbearing [1–3]; and the early postpartum period is a particularly risky time for first and recurrent episodes of severe mental illness [4, 5]. This heightened risk of mental illness or mental health difficulties may go some way to explaining why maternal suicide is the leading direct cause of death between six weeks and a year after the end of pregnancy and the second most frequent cause of death during or within six weeks of the end of pregnancy in the UK and Ireland [6]. Furthermore, a meta-analysis of 14 studies found the worldwide prevalence of suicide attempts during pregnancy was 680 per 100,000 and 210 per 100,000 during the postpartum period [7], and the prevalence of self-harm ideation during the perinatal period was found to range from 5 to 14% [8]. As we can assume even greater numbers of mothers experience suicidal ideation during the perinatal period, the development of low cost, accessible and effective methods to identify and treat mothers experiencing suicidal ideation and engaging in suicidal behaviour is essential.

Several psychological and psychosocial factors have been found to be associated with increased risk of suicidal ideation and behaviour during the perinatal period. In their systematic review, Reid et al. [9] identified 59 quantitative studies that investigated associations between psychological and psychosocial risk factors and suicide outcomes in pregnant and postpartum mothers. The authors found strong evidence that abuse experienced at any point in a woman’s life was associated with suicidal ideation, attempts and death, and that a lack of social support was significantly associated with suicidal behaviour. Although these risk factors indicate mothers who might be more at risk of suicide, they do not tell us what might trigger and maintain suicidal thoughts and very few of the studies in Reid et al.’s [9] review provided a rationale for why those particular factors were investigated.

Qualitative research with key stakeholders of perinatal suicide (e.g., mothers who experience suicidal thoughts and perinatal health care professionals) offers the flexibility and scope to investigate factors that may trigger and sustain suicidal ideation and behaviour, identify novel factors, as well as investigate how best to support mothers experiencing suicidal ideation and behaviour during the perinatal period. A meta-synthesis of eight qualitative studies investigated the lived experiences specific to suicidal ideation of mothers with postpartum depression [10]. Six themes were identified, which described mothers hiding their suicidal feelings to adhere to the cultural expectations of motherhood, incongruence between the expectations and reality of motherhood, loss of control, the overwhelm of motherhood on top of pre-birth responsibilities, lack of sleep and social support as a buffer to suicidal thoughts. These findings offered initial insights into some of the possible drivers of suicidal ideation when diagnosed with postpartum depression; however, they did not suggest how these factors might interact to trigger suicidal ideation and behaviour. As part of a grounded theory study of twelve mothers who experienced suicidal thoughts, and some who also attempted suicide, Reid et al. [11] developed a model of psychological factors that culminated in a mother experiencing suicidal thoughts and then making a suicide attempt during the perinatal period. According to this model, the process involved mothers feeling attacked by motherhood which led to feeling like a failure, self-identifying as a *“bad mother”* [11](p.12) and subsequent appraisals of entrapment and/or defeat. Suicidal behaviour became an increasingly appealing option when nothing resolved the distress and as mothers collated reasons for why they perceived they needed to die. Mothers might then act on their thoughts when they entered a state of intense darkness brought on by a trigger, followed by a lapse in the conflict between the desire to live and desire to die coupled with an opportunity to attempt suicide.

Reid et al.’s [11] model furthers our theoretical understanding of suicidal ideation and behaviour during the perinatal period from the perspective of mothers, but perinatal suicide is an area of research that is incredibly complex and there are many elements (e.g., a mother’s environment, physical health, obstetric experience) that have the potential to contribute to maternal suicidal ideation and behaviour [12, 13]. Alderdice [14] describes perinatal mental health research, policy and practice as a *“balancing act of identifying what is unique to women in the perinatal period and what is similar to mental health in the general population”* (p. 111).With regards to perinatal suicide, we can argue that this goes one step further and it is also important to tease out what is unique to suicidal mothers and what is similar to non-suicidal mothers experiencing mental health difficulties. Conducting qualitative research with healthcare professionals, who work directly with suicidal mothers during the perinatal period, can provide insights developed through collective observations of working with mothers who experience varying levels of suicidality as well as mothers who do not experience suicidal ideation. These insights are crucial to understanding current practices and how the identification of mothers experiencing perinatal suicidal ideation and interventions offered to reduce and prevent suicidal ideation and behaviour can be improved.

Previous research with healthcare professionals who work with suicidal patients has focused on how equipped they felt to deal with suicidal behaviour [15–17]. However, very few studies have focused on healthcare professionals who work with mothers experiencing suicidal ideation and behaviour during the perinatal period. In their cross-sectional study of 95 Australian midwives and 86 maternal child health nurses (similar to health visitors in the UK), Lau et al. [18] noted that maternal child health nurses had more positive attitudes towards perinatal suicide prevention than midwives. However, the authors did not investigate how professionals prevented suicide or how professionals identified mothers at risk of suicide during the perinatal period. In addition, they focused on healthcare professionals working with all mothers rather than those who work with mothers experiencing mental health difficulties specifically.

Through interviewing a varied sample of mental health professionals who worked directly with mothers who experienced suicidal ideation and behaviour during the perinatal period while under their care, we anticipated gaining a holistic insight into the factors that are perceived as being associated with the development of suicidal ideation as well as what the professionals perceived as beneficial in the treatment of these mothers. To that end, we aimed at exploring the experiences and perceptions of mental health professionals who have worked with suicidal mothers during the perinatal period.

## Method

### Design

The qualitative study employed a cross-sectional design to capture experiences and perceptions of perinatal mental health staff who have worked with mothers who were suicidal during pregnancy and/or the first twelve months following birth.

### Ethical considerations

The study was reviewed by a National Health Service (NHS) Research Ethics Committee (18/NW/0849) and received ethical approval from the Health Research Authority. Each participant provided informed consent and a specifically developed distress management protocol was followed if a participant became upset or distressed during the interview or any contact with the researcher. On completion of the interview, all participants were provided with information for further mental health support and asked if they would like to receive a summary of the findings.

### Participant inclusion and exclusion criteria

Participants were perinatal mental health professionals who had direct contact with and provided care to mothers who experienced suicidal ideation and/or behaviour during the perinatal period. This participant group included consultant and trainee psychiatrists, clinical psychologists, mental health nurses and nursery nurses working in inpatient or community services (i.e., psychiatric inpatient Mother and Baby Units [MBUs] and Perinatal Community Mental Health Teams [PCMHTs]) in the UK. Professionals were required to have been in their post for at least three months to ensure they had sufficient professional experience to answer the interview questions. Non-permanent staff, such as agency staff and those providing short-term cover, were excluded due to their varying levels of professional experience within perinatal mental health services.

### Data collection and procedure

The study was advertised via email circulated to professionals working within an MBU and PCMHTs in the Northwest of England. Advertisements were also posted on Twitter (now known as ‘X’) to recruit professionals from services across the UK. All potential participants who enquired about taking part were provided a participant information sheet. Once potential participants had had the opportunity to read the information sheet, had all questions answered by HER and remained willing to take part, they completed a consent form. HER interviewed all participants and administered a demographic questionnaire which asked for their ethnicity, gender, years since qualification, and information regarding their post and the service within which they worked. We developed a semi-structured interview schedule by combining our experiences of working, and conducting research, with women during the perinatal period. The interview schedule was intentionally designed to be flexible, with open questions, to allow the participants to describe their experiences and perceptions at length and to encourage them to talk about anything pertinent to the research question. The interview schedule was reviewed by a perinatal mental health professional and a previously suicidal perinatal mental health service user in the design stages of the study to ensure the schedule was suitable, appropriate, and covered all the necessary areas to meet the aims of the study. Interviews were conducted face-to-face in a private room at the participant’s place of work or via the telephone, depending on their preference. All interviews were recorded using an encrypted dictaphone and were transcribed verbatim, eight by HER and seven by a professional transcription service.

### Analysis

Reflexive thematic analysis [19, 20] was used for data analysis. Guided by Braun and Clarke’s [19, 21, 22] six-phase-process to doing thematic analysis, we aimed to produce a deep and compelling interpretation of the data. Reflexive thematic analysis allowed us to achieve this because, unlike coding reliability and codebook thematic analyses, it is a method that embraces the subjectivity of coding, gives the researcher(s) an active role in constructing themes and space to reflect on the analysis [23]. The six-phase-process was conducted by HER under the supervision of DE, DP and AW. The analysis was undertaken by hand using a pen and paper (i.e., qualitative data analysis software was not used). The analysis process firstly involved HER familiarising herself with the data through transcribing, reading and re-reading hard copies of the interview transcripts to help with the identification of data relevant to the research question; at this stage potentially interesting excerpts as well as any observations of initial trends were noted. The second phase saw the generation of initial codes whereby interesting and informative data items were highlighted. Regular team meetings saw HER discuss her coding progress with DE, DP and AW. Once coding was completed, the third phase involved HER reviewing the coded data and then combining codes according to their shared meaning to generate themes. HER discussed this theme generation with DE, DP and AW who made analytic suggestions, as well as posed questions related to the developing themes to aid the organisation of the themes. Next, these potential themes were reviewed, firstly by HER and then by DE, DP and AW to ensure coherence of each theme, that there were adequate data to support each theme, and that each theme was meaningful and informative with regards to reflecting the dataset and answering the research question. Phase five involved defining and naming the themes and sub-themes and choosing the extracts of the interviews for inclusion in the report write-up. This fifth phase was firstly conducted by HER who sought guidance from DE, DP and AW when required. The final phase involved HER writing the first draft of this report and making final revisions to the themes and sub-themes following feedback from DE, DP and AW. Coding had both a semantic (i.e., coding explicitly stated ideas) and latent (i.e., coding of more implicit ideas underlying what is explicitly said) focus [24]. The analysis was predominantly inductive (i.e., codes were produced to reflect the content of the data), although we acknowledge coding will have been partially deductive to ensure the codes and themes provided a meaningful contribution to meeting the research aims [22].

### Philosophical underpinning

A contextualist epistemology was adopted which posits that how participants put their experiences into words, provides access to their version of reality, and enabled us to acknowledge how participants’ experiences were socially mediated [25]. For example, the analysis explored how professional roles affected experiences and perceptions of working with mothers who are suicidal. Aiming to describe completeness rather than convergence of responses is another key proponent of the contextualist epistemology and this approach allowed us to include unique responses rather than agreement between participants only [26]. To focus on the patterns of meaning ascribed by participants, we took an experiential orientation to data interpretation which allowed us to prioritise professionals’ own accounts of their experiences and perceptions, rather than explore the socio-cultural factors that contributed to participants’ accounts [24, 27].

### Positionality

#### Position within the data

HER, a PhD student and mother, had experience of working alongside perinatal mental health professionals on an MBU in her previous research work and she also had experience of interviewing maternity healthcare professionals (e.g., [28, 29]. DE was a professor of mental health and inclusivity and a mother whose research has examined maternal mental health in community, clinical, and forensic settings. DP was a senior clinical lecturer, clinical psychologist and father with expertise in the development and evaluation of suicide prevention interventions. DP had extensive clinical experience of delivering, and supervising the delivery of, psychological therapy to individuals experiencing suicidal ideation and behaviour across community, mental health inpatient and forensic settings. AW, a senior lecturer, clinical psychologist and mother, had extensive clinical and research experience of and expertise in perinatal mental health using qualitative and quantitative research. In addition, through her clinical work in an MBU, AW had direct experience of supporting mothers with suicidal experiences and working with a perinatal mental health multi-disciplinary team.

#### Reflexive judgement

All authors met regularly to discuss sampling, recruitment methods, coding and theme development. The diversity within our author team with regards to clinical background, experience of working with perinatal mothers and experience of conducting qualitative research, allowed us to challenge each other’s assumptions and pre-conceptions when collecting and analysing the data. Our collective clinical and research expertise allowed the team to focus on what might set apart professionals’ experiences of perinatal mothers from working with other patient groups who experience suicidal thoughts and behaviours.

## Results

### Participant characteristics

Between May 2019 and January 2020, 18 professionals expressed an interest in participating, HER lost contact with three potential participants, therefore a final sample of 15 eligible professionals were recruited and interviewed. The sample size and varied characteristics of the sample in terms of professional posts, length of time in post, gender, ethnicity and place of work, was practicably achieved within the time- and resource-constraints of the study, with the sample generating sufficient data to answer the research question [30]. It should be noted that all interviews were conducted prior to the outbreak of Covid-19 in the UK and associated restrictions being imposed in March 2020. Interviews ranged in length from 24 min to 1 h 16 min.

We recruited four consultant psychiatrists, one trainee psychiatrist, three clinical psychologists, one community operational services manager, one ward manager, two staff nurses, one nursery nurse and two occupational therapists. Twelve participants were White British, two were mixed White and Black Caribbean and one participant was British Indian. Eleven participants were female and four were male. Eight of the participants worked in an MBU and seven worked in PCMHTs at the time of the interview and all participants were recruited from across England. The time since the participants qualified for their role ranged from two years to 32 years.

### Findings

**Fig. 1:**
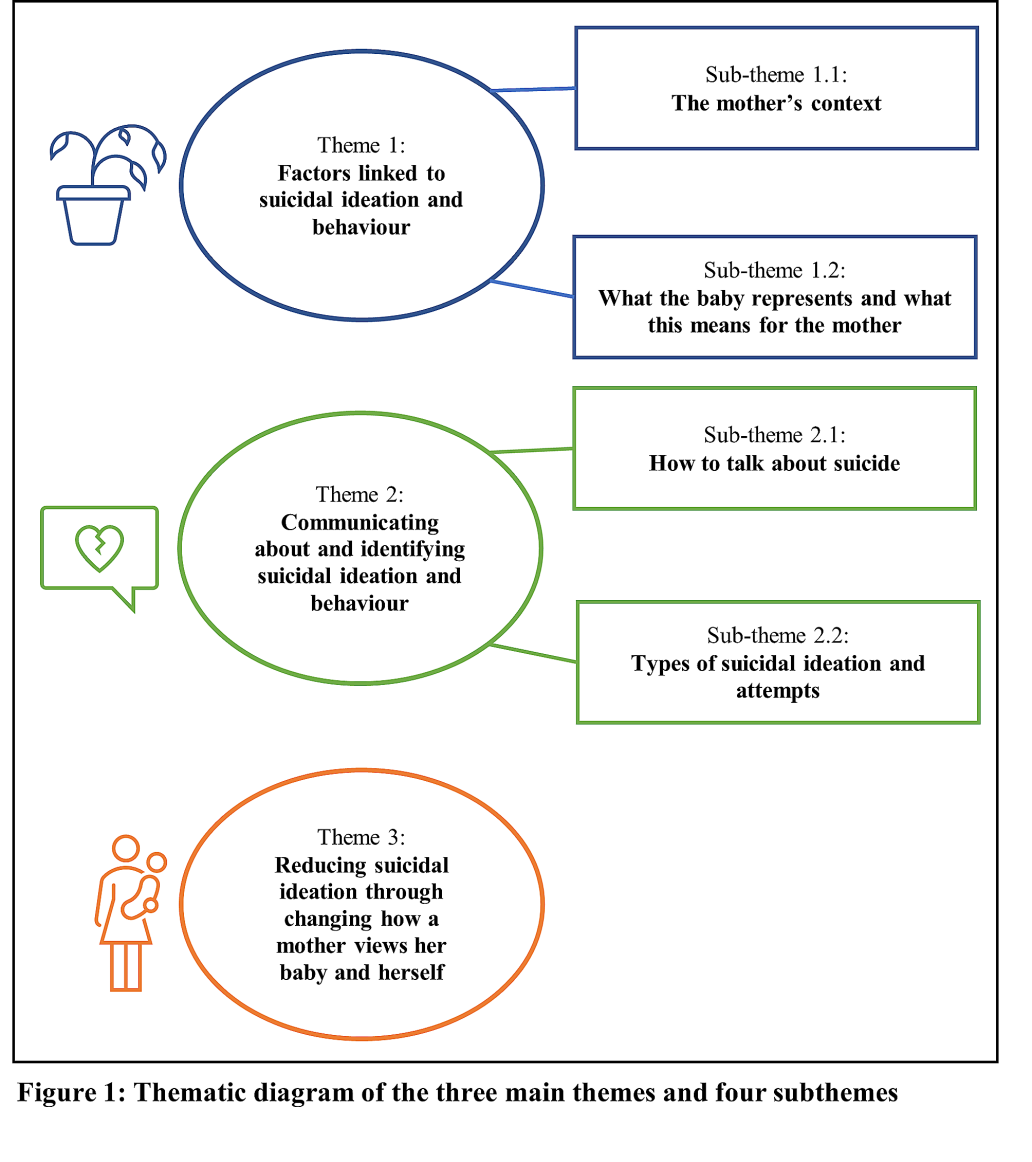
Q1Thematic diagram of the three main themes and four subthemes

Following analysis, three main themes and four sub-themes were developed from the interview data (see Fig. 1 for the thematic diagram). All participants provided data for each main theme, although diversity of experiences and perceptions are reported in line with the contextualist approach to analysis.

### Theme 1: factors linked to suicidal ideation and behaviour

All 15 professionals discussed which factors they assessed or deemed contributory in relation to suicidal ideation and behaviour when a woman was under their care. These factors might be those that professionals asked about as part of a formal assessment of suicide risk (i.e., by psychiatrists during their initial meeting with mothers referred to their care). In addition, other professionals, who were not required to carry out such formal assessments (e.g., nursery nurses), provided an insight through their daily duties into the warning signs or problems shown by mothers experiencing suicidal ideation and behaviour. We developed two sub-themes under this theme: (1.1) *the mother’s context* and (1.2) *what the baby represents and what this means for the mother*.

### Sub-theme 1.1: the mother’s context

When working with mothers who experienced suicidal ideation/behaviour, the role of social support was deemed an important yet complex factor. Professionals highlighted that social support during the perinatal period was essential because, during this period, mothers were physically vulnerable, childcare was psychologically demanding and often a mother required practical assistance with day-to-day household tasks (e.g., shopping and cleaning), while she cared for the baby. As most mothers relied on their partner for support, professionals identified that the ending or straining of romantic relationships could have a significant effect on a mother’s suicidal ideation/behaviour:

*“…where relationships have broken down– often that can be a really important part of a mum developing that sense of hopelessness and helplessness because parenting is such a relentless job, and actually having no-one to support you and share it with is really hard”* (Inpatient Clinical Psychologist).

In contrast, professionals observed that some social support, although well-intentioned, could be overbearing and made the mother feel redundant, especially when others insisted on carrying out the childcare duties (e.g., changing nappies, feeding, and settling):

*“…you allow people to take over. And then you feel despondent about your role as a mother, that you’re not doing the care. And then people are reinforcing those thoughts by saying to you, ‘you’re not doing it right’ […] makes you feel worthless as a mum”* (Community Operational Services Manager).

Professionals expressed awareness of some mothers who evaluated who else could look after their child(ren) if they were to die by suicide and therefore these mothers might not perceive helpful social support (with regards to childcare) as a factor protecting them from attempting suicide:

*“If they know that there’s not many people around to look after the children if they were to kill themselves, that’s a protective factor because they’re like ‘who else would look after them?’”* (Community Consultant Psychiatrist 1).

A mother’s experience of her own childhood, particularly if she experienced abuse, was also reported by professionals as an important aspect of what could impact a mother’s suicidal ideation/behaviour. In particular, clinical psychologists reported that mothers who were victims of childhood abuse often feared their own children would be at risk of abuse, which seemed to contribute to thoughts of suicide or infanticide because these mothers thought of sparing their baby:

*“If they’ve had parents who have been abusive and they believe that ‘therefore I’m at risk of being abusive’ […] then there could be a risk there of just feeling not good enough for the baby and therefore wanting to end their life or the baby’s”* (Community Clinical Psychologist 2).

Whether the pregnancy was unwanted was also deemed a major factor in relation to a mother’s suicide risk. Professionals theorised that the feeling of being trapped in the pregnancy was the reason for considering suicide as an option to escape pregnancy, which appeared to be a more salient factor for mothers who presented to services after 24 weeks of pregnancy and consequently were unable to have a termination:

*“[she is] pregnant and doesn’t want the baby, but is too far ahead in the pregnancy for a termination […] she is now feeling stuck, and is saying that if she can’t have a termination she’s going to kill herself”* (Inpatient Consultant Psychiatrist 1).

### Sub-theme 1.2: what the baby represents and what this means for the mother

Professionals’ narratives revealed that the way a mother perceived their baby was an especially important factor in mother’s experiencing suicidal ideation. All professionals stressed that they paid particular attention to how a mother interacted with and conveyed feelings towards her baby. Psychiatry and psychology staff described that they examined the mother-baby-relationship in relation to the mother’s mental health difficulties, whereas nursing staff and occupational therapists were more focused on what they could do to improve this relationship. During interviews, when professionals were prompted to reflect on whether the baby was a protective factor against suicide for a mother, it became apparent that professionals viewed protectiveness as an oversimplification of the mother-baby relationship but found identifying what the baby represented for mothers experiencing suicidal ideation more helpful. According to professionals, a mother’s experience of the birth, of breastfeeding, of providing childcare and also how she interpreted the baby’s cues were important factors that contributed to what the baby represented for the mother. If a mother perceived the birth as traumatic, the mother might blame the baby for her trauma, which made fostering a secure and positive bond with the baby particularly difficult and this unstable bond might result in a woman feeling as though others would be better at caring for the baby:

*“… that baby resulted in a lot of hurt for you, that must be really difficult to disregard, and you know, I think intrinsically as humans I think that when we get hurt we blame other people”* (Community Operational Services Manager).

Many professionals raised breastfeeding as an activity that carried particular weight for mothers’ feelings of adequacy which could then trigger suicidal thoughts. Professionals spoke about how breastfeeding meant *“the baby needed her for that biological purpose […] the thing that was keeping her here”* (Inpatient staff nurse 1). As this role could not be carried out by anybody else, apart from the mother, breastfeeding could be a protective factor for mothers. However, if a mother was not meeting her breastfeeding goals, this inability to meet one of her baby’s fundamental needs could make her feel inadequate:

*“Lots of women put high expectations on themselves to breastfeed [.] So [there is] this idea of, ‘I’ve got one job, and I can’t do that’”* (Community Clinical Psychologist 2).

Comparable to this feeling of inadequacy, professionals frequently reported that the baby represented a sense of failure for the mother and a perceived reminder that she was not good enough to be a mother. These feelings could also be reflected in a mother’s behaviour towards her baby which would exacerbate her feelings of failure, as expressed by this professional:

*“She found the experience of providing childcare quite shameful because she thought she was no good at it […] And it was kind of one of those self-fulfilling prophecies because she did it really superficially and minimally, and so didn’t get an awful lot of feedback from her son, and so felt like that just confirmed that she was no good at it, and she shouldn’t be doing it, and she might as well die because someone else could care for her son a lot better than she could”* (Inpatient Clinical Psychologist).

Professionals’ accounts indicated that a mother’s interpretations of her baby’s behaviour and how she compared her interactions with baby to the baby’s interactions with others was pertinent. In addition, participants expressed that the risk of suicide was raised in mothers who lacked confidence in parenting or knowledge of baby behaviour. In one professional’s example, a mother might use the baby’s temperament to shape her beliefs of how the baby feels about her:

*“They might sort of assign things to the baby that wouldn’t normally be assigned […] that ‘she doesn’t like me or, you know, she’s just doing this to be difficult for me. She’s fine with other people. But this is, you know, specifically cos she doesn’t like me’. So, it depends on how they interpret the difficult temperament”* (Community Consultant Psychiatrist 1).

### Theme 2: communicating about and identifying suicidal ideation and behaviour

This main theme encapsulated how participants enquired about suicidal ideation and behaviour with mothers and describes the different types of suicidal experiences that professionals identified. Across the dataset we identified that how professionals talked to mothers about their suicidal thoughts and behaviour was influenced by how professionals contextualised these discussions, and so we developed sub-theme: *(2.1) how to talk about suicide*. Sub-theme *(2.2) types of suicidal ideation and attempts* describes the variation in suicidal thoughts reported by mothers and suicide attempts observed by professionals.

### Sub-theme 2.1: how to talk about suicide

Professionals needed to encourage mothers to disclose their thoughts and behaviour, understand why mothers experience or engage in these thoughts and behaviour, and support the mother’s developing awareness of her own suicide risk. Three aspects for facilitating these conversations were identified from professionals’ narratives: (1) ensuring they understood the context in which the mother was asked to discuss her suicidal ideation and behaviour, (2) establishing a trusting relationship with the mother, and (3) being alert to non-verbal communication.

Regarding the context that surrounded interactions with mothers to enquire about their suicidal ideation and behaviour, professionals perceived the main concern that mothers had about engaging in these conversations was the possible involvement of social services and the consequences of this involvement:

*“Often this conception is that we’re in league with social services and there’s an agenda to take away their baby. So, if there’s any hint that the woman’s sort of maybe carrying that anxiety, I would address it sort of at the beginning. And then usually you can tell that they get a bit more relaxed”* (Community Consultant Psychiatrist 1).

Prior to professionals enquiring about suicidal ideation and behaviour, nursing staff and occupational therapists reported how they tried to identify commonalities with a mother to facilitate her engagement and build a trusting therapeutic relationship. In contrast, psychiatrists revealed that they tended to ask lots of informal questions to draw the mother into a conversation about her baby as a way of engaging her. All professionals acknowledged that mothers required communication to be personalised to their needs and personality. They also agreed that asking a mother outright about her suicidal ideation and behaviour did not always facilitate their assessment. Thus, they used different questions to assist with their enquiry of suicidal ideation and behaviour:

*“[I ask] when they’re at their lowest, like what does that look like? […] If I was a fly inside your head, what would I be watching or seeing?”* (Community Clinical Psychologist 2).

Not only did professionals need to initiate this particular enquiry, but they also had to be alert to mothers trying to use non-verbal communication to convey their suicidal thoughts and intentions:

*“One of our teams went out to assess somebody and they found a noose had been tied, hanging from the ceiling […] that is a communication in a way. Like somebody leaving something like that out, may not say to you ‘I’m feeling suicidal’, but they know you’re coming round and it’s a way of communicating isn’t it, it’s ‘I feel so distressed, but maybe I can’t talk about it’”* (Community Clinical Psychologist 1).

### Sub-theme 2.2: types of suicidal ideation and attempts

In the interviews, it became apparent that professionals worked with mothers who experienced a range of different thoughts that could all be classed as suicidal ideation. The data also revealed that professionals took all types of ideation seriously. According to professionals, suicidal thoughts in mothers ranged in severity from fleeting thoughts (i.e., *“[some] describe the suicidal thoughts as more being kind of intrusive thoughts”*,Community Consultant Psychiatrist 2) to passive suicidal ideation of wanting to vanish (i.e., *“[a mother might say] I’m just going to pack my bags and disappear and that would be the best thing all around for everyone”*, Community Occupational Therapist) and, finally, to preparatory thoughts to plan a suicide attempt (i.e., *“they’ve kind of kept all their medication […] it’s in the back of their mind that they have got something if they wanted to”*, Ward Manager). Some professionals also identified a *“dark despair moment”* (Community Operational Services Manager), when suicidal ideation was experienced as incredibly intense by mothers, and professionals saw this as the moment when a suicide attempt was most likely to happen. Professionals described this intense ideation as a stage whereby a mother’s focus was on herself dying and anything that was previously preventing her from dying was forgotten or avoided:

*“They sort of switch off from everything around them […] And any protective factors have gone […] So nothing can take them away from that thought that they want to hurt themselves”* (Inpatient Nursery Nurse).

Professionals also reported that suicide attempts could be interpreted as ranging in seriousness depending on the method used and the setting. For example, some professionals viewed a suicide attempt on an inpatient unit as a form of communication because of the likelihood of it being noticed by staff and then interrupted:

*“…that people might say ‘well, she’s attention-seeking’, but from my point of view, she’s asking for help for some reason […] she attempted to hang herself in the shower, but all our shower rails are ligature risk-assessed and they fall down if you put any weight on them. So, it’s really difficult to assess well was she actually trying to hang herself? Did she know that it would break?”* (Inpatient Occupational Therapist).

Similarly, some professionals also spoke about mothers who felt unsure about attempting suicide which might influence the method used and that mothers appeared to consider the consequences of attempting up until and even during the attempt.

*“There might be a level of conscious or unconscious ambivalence that says, kind of yeah, take 16 tablets, but don’t take anymore and that way you’ve tried to die”* (Inpatient Clinical Psychologist).

Contrastingly, most professionals stressed that the majority of mothers tended to attempt suicide using violent methods (e.g., jumping from a height and hanging) during the perinatal period. Some professionals pondered if one reason for this use of violent means was the mother’s lack of consideration of the consequences prior to the attempt:

“*By doing it by such violent means it’s kind of like you haven’t got the time to have even reflected on ‘is this the right thing to do?’”* (Community Operational Services Manager).

One professional proposed that self-punishment might guide a mother to choose a more violent method of suicide attempt, perhaps due to the shame and guilt associated with feeling suicidal during the perinatal period:

*“I also just get the impression that it’s something about somehow punishment. Like they deserve to die in this sort of like, quite brutal way”* (Community Occupational Therapist).

### Theme 3: reducing suicidal ideation through changing how a mother views her baby and herself

This final theme reflects the striking pattern of participant responses naming the support that helped mothers reframe the way they viewed their baby and in turn themselves. Given professionals highlighted an association between a mother’s perception of the baby and a mother’s suicidal ideation and behaviour, it follows that professionals also believed that changing that perception held by mothers was important to reduce their suicidal thoughts. This changing of perception was highlighted particularly by the psychiatrists and psychologists interviewed for this study. These professionals emphasised a mother’s unique purpose and her importance to her baby, challenged the mother’s perception that she was replaceable and highlighted how the baby’s bond would grow, despite the mother feeling inadequate. One clinical psychologist provided a detailed example of how they demonstrated a mother’s importance to her baby:

*“If you weren’t here, what would– how would it feel for your baby? What would it be like for your baby growing up without a mum? How do you think your baby will be able to talk about you not being here? […] every time you turn around and you’re, you know, quite often see your baby looking for you, what’s your baby kind of thinking and feeling? Why would the baby look for you as opposed to anybody else?”* (Community Clinical Psychologist 2).

Similarly, strengthening the mother-baby bond through changing a mother’s negative perception of her own baby’s view of her was a key focus for all professionals when working with mothers experiencing suicidal ideation:

*“Interventions like VIG [video interactive guidance] and things you can see the difference that those make with mums, where they thought they didn’t have a good relationship, where they thought baby didn’t like them, that baby hated them […] you can see the difference and the mum realising that actually, they do have a good relationship with baby”* (Inpatient Occupational Therapist).

Lastly, participants highlighted that increasing a mother’s confidence at performing the tasks of motherhood was key to combatting feelings of inadequacy as a mother and in turn, reduce suicidal ideation:

*“Through practice she was able to do bits and bobs and getting the reaction from him, the more she felt she was doing an okay job as a mum, and the more her mood lifted and her suicidality reduced”* (Inpatient Clinical Psychologist).

We also noted that this professional described a mother’s confidence increasing through her being *able* to do tasks; this suggests a mother does not need to necessarily excel at mothering but just feeling able to mother could potentially reduce suicidal ideation. Furthermore, this quote highlighted the importance of perception rather than performance; a mother feeling as though she is doing a good enough job mattered more than actually doing a good job.

## Discussion

This novel qualitative investigation of the experiences and perceptions of mental health professionals is the first to have used reflexive thematic analysis to analyse the accounts of healthcare professionals providing direct care to suicidal mothers during the perinatal period. The sphere of perinatal suicide research is incredibly complex and nuanced and the myriad potential factors that might contribute to maternal suicidal ideation and behaviour is currently not well understood. The professionals’ accounts collected in this study provide potential factors that should be considered and investigated further to improve clinical practices. We developed three main themes which outlined factors that professionals perceived as important for the detection and reduction of suicidal ideation and behaviour.

In the current study, health professionals perceived mothers’ perceptions of social support as an important factor relating to a mother’s suicidal ideation and behaviour. Typically, support perceived as low-quality or lack of any social support has been found to increase the likelihood of mental health difficulties and suicidal behaviour in mothers [9, 31, 32]. One novel finding of this study is the professionals’ descriptions of overbearing social support which excluded the mother and minimised her role as the primary caregiver, but also demonstrated that the baby could be well cared for if the mother died. This concerning combination, which could enable a mother to attempt suicide, has not yet been reported elsewhere in the literature but it highlights the complexities of social support and how it is perceived, both by mothers themselves and the healthcare staff offering support.

Professionals highlighted the importance of breastfeeding for some mothers experiencing suicidal ideation. The important impact of perceived breastfeeding success or failure on a mother’s thoughts, feelings and beliefs has been echoed in previous literature. Brown [33] reported that breastfeeding is seen as a central part of becoming a mother and therefore mothers expect to be able to meet their breastfeeding goals. When a mother cannot meet these goals, she can experience *“shattered expectations”* [34, p.4] and feel of *“no use to her baby”* [34, p.5], but ‘successful’ breastfeeding can be a source of pride, pleasure and achievement [33]. The lack of evidence about breastfeeding intention, initiation and duration and suicidal outcomes coupled with the well-documented complex relationship between breastfeeding and maternal mental health [35–38], indicates that this would be an important direction for future research. Although we must exercise caution when interpreting the transferability of our qualitative findings, we propose that a mother’s feeling of uselessness, perhaps coupled with a strong internal or external pressure to breastfeed, could offer a potential mechanism for why not meeting breastfeeding goals for some mothers might drive suicidal ideation. Similarly, professionals also highlighted that mothers who lacked confidence in their parenting abilities were more likely to experience suicidal ideation or behaviour. Low self-efficacy has been correlated with suicidal ideation during the postpartum period [39]. Maternal self-efficacy had been identified as a mediator of infant difficulty and maternal depression [40] and a mediator of mothering competence and maternal depression [41], indicating that maternal self-efficacy is important for coping with the challenges of early motherhood. However, how low self-efficacy might be implicated in the development or maintenance of a mother’s suicidal thoughts is not understood but has implications as a potential treatment target, and therefore determining the potential role of self-efficacy on the development of maternal suicidal ideation and behaviour should be a focus of future research.

Professionals reported several ways that they used and believed to encourage mothers to disclose their suicidal thoughts and behaviours, such as personalising communication to the needs of the mother and addressing any concerns a mother might have about social services involvement. However, this study also found professionals did not feel direct questioning always facilitated assessments of suicidal ideation and behaviour. This finding contradicts research and guidance that advocate consistent use of direct questioning when assessing suicidal thoughts, plans and intent (e.g., 42, 43). Furthermore, professionals in this study reported that some mothers were hesitant to verbally communicate their suicidal intent, which should heighten the need for direct conversations about suicide to ensure professionals have an accurate understanding of a mother’s suicidal thoughts and behaviour. Despite this, professionals were keen to stress that they tailored their approach to enquiring about suicide with the intention of encouraging a mother to disclose her thoughts and behaviours which can be complex when some mothers will want to conceal their intentions [44] and the nature of suicidal ideation can change rapidly [45]. Moreover, more honest disclosure of suicidal thoughts has been observed when health providers focus on building a trusting relationship [46, 47] which was also deemed a priority by our sample.

With regards to types of suicidal ideation and attempts, participants reported that mothers could experience a moment of intense darkness and this finding echoes the acute period of suicidal ideation whereby suicidal desire becomes incredibly intense (see *“the darkness descends”* in Reid et al. [11, p.15]). In the current study, professionals also suggested that some mothers might attempt suicide with hesitation which on the surface seemed to contradict this idea that mothers feel an intense need to die before attempting to harm themselves. Research with Western-European adults suggests that those who attempted suicide could be clustered into three different groups: *“impulsive-ambivalent”*, *“well-planned”* and *“frequent”* [43, p.1]. Those adults in the *“impulsive-ambivalent”* cluster appeared to make less lethal and serious attempts, whereas those in the *“well-planned”* group carefully planned their suicide attempt and often used alcohol or drugs to impair their judgement prior to attempting. Further possible insights into influences over how an individual attempts suicide might be drawn from a study using a monetary rewards task with a sample of adults aged 60 years and older [48]. The authors proposed that suicide *“can be viewed, in some cases, as an attempt to obtain immediate relief while foregoing all future rewards”* [48, p. 138] and they hypothesized that less serious suicide attempts would be made by individuals who prefer immediate rewards whereas those who are more patient would carry out the most serious attempts. Their study compared five groups of participants: the first group had made a high-lethality suicide attempt, the second group had made a low-lethality attempt, the third experienced suicidal ideation only, the fourth were depressed but not suicidal and the fifth were controls with no history of any psychiatric disorder. Participants completed Kirby’s Monetary Choice Questionnaire [49], a delay discounting task that presents 27 choices between monetary rewards that are small and immediate or larger and delayed. The authors found that those who made high-lethality suicide attempts were more willing to delay future rewards whereas those who made low-lethality attempts were more impulsive in their preference for immediate rewards. Although Dombrovski et al.’s [48] study focused on older adults, analogous to perinatal mothers, older adults appear to engage more in high-lethality suicidal behaviour [50–53]. Therefore, taking Fiedorowicz et al.’s [43] and Dombrovski et al’s [48] findings together, we could theorise that when mothers enter a period of intense darkness prior to an attempt (as identified by the current study) the seriousness and lethality of the attempt that follows could be determined by an individual’s planning, impulsivity and choice behaviour.

Strengthening the mother-baby bond was deemed an important way of combatting a mother’s feelings of inadequacy when experiencing suicidal ideation, by professionals. Previous studies have identified that mothers who perceived having an impaired bond with their baby at six to nine months postpartum were more likely to have suicidal ideation [54] and mothers classed as ‘highly suicidal’ were observed as demonstrating less sensitivity and reciprocity during mother-infant interactions [55]. The mechanism that links bonding and maternal suicidal ideation and behaviour is unclear and currently there is no research on whether interventions that improve bonding (perceived and observed) also reduce suicidal ideation and behaviour. However, studies have been conducted to establish whether improving mother-infant bonding can also improve mental health difficulties. Loh et al. [56] undertook an observational study of the effectiveness of the *Sure Mums* intervention which aims to improve mother-infant bonding. In a sample of 25 mothers, self-reported scores on the *Postpartum Bonding Questionnaire* [57], *Edinburgh Postnatal Depression Scale* [58] and *Global Assessment of Functioning* [59] all significantly improved post-intervention. Furthermore, a systematic review which evaluated 19 studies of interventions for postpartum depression that also assessed mother-infant interactions reported that interventions which focused on the quality of the dyad relationship had the greatest efficacy at reducing symptoms of postpartum depression [60]. For suicide prevention interventions to be effective they need to target suicide outcomes directly, and therefore moving forward, the impact of interventions that aim to improve mother-infant bonding during the perinatal period upon suicidal outcomes should be investigated.

### Strengths and limitations

Strengths of this study include the recruitment of participants working within both inpatient and community perinatal mental health services from several different NHS Trusts across England, and therefore serving varied communities of mothers. We also recruited a range of healthcare professionals to gain perspectives from the multiple disciplines providing perinatal mental health care.

Despite the data collected from the sample of 15 professionals proving sufficient to answer the research aim within the pragmatic constraints of the research, the findings have limited transferability due to the relatively small sample size and because professionals only worked in England. We deliberately did not include maternity healthcare professionals, such as midwives and health visitors, in our sample because we wanted to ensure participants had experience of regularly working with suicidal mothers, hence we limited our sample to perinatal mental health professionals. We should also highlight that, fortunately, very few professionals interviewed for this study had experience of working with mothers who had gone on to die by suicide, which meant we were unable to draw any major conclusions about mothers who fatally attempt suicide.

### Implications and future research

Our findings have implications for professionals working with mothers during the perinatal period in England and for the content of the training these professionals receive. Firstly, we suggest that professionals should be vigilant to mothers who receive support that could, albeit unintentionally, exclude her as the primary caregiver because this could trigger feelings of worthlessness and increase the likelihood of suicidal ideation and behaviour. Observations of this kind of support should be explored further with the mother. Secondly, professional training for those who are required to ask mothers about suicidal ideation and behaviour during the perinatal period should stress the importance of enquiring about suicidal ideation and behaviour in a sensitive and responsive way and emphasise that mothers might fear losing custody of their child(ren) if they disclose mental health difficulties; if this is a concern, professionals should then address these fears and re-frame the enquiry as a means to ensure appropriate support is offered. Thirdly, training should also educate professionals of the variation in how maternal suicidal ideation could manifest when they enquire about suicidal thoughts (e.g., *“sometimes I want to disappear”* constitutes passive suicidal ideation) and to respond to these varying manifestations seriously.

Although our findings do not provide direct evidence to support the existence of a mechanism(s) linking self-efficacy or breastfeeding goals to suicidal ideation and behaviour, the findings provide an indication that these factors should be investigated in the future. Furthermore, providing support to mothers that promotes a mother’s strengths and builds her confidence in caring for her baby as well as supporting breastfeeding should benefit all mothers. Determining these psychological mechanisms of perinatal suicide in future research have the potential to impact the detection of suicidal mothers and prevention and reduction in suicide outcomes in mothers globally. Thus, future research should seek to further our understanding of self-efficacy and ‘successful’ breastfeeding on suicidal outcomes for mothers. Due to the importance of changing how a mother viewed her baby and herself to help reduce suicidal ideation, an area warranting further research should be the extent to which interventions to strengthen mother-infant bonding, especially those that emphasise a mother’s unique importance for the baby, impact suicidal ideation and behaviour.

Many other health professionals who work with mothers during the perinatal period are likely to encounter mothers experiencing suicidal ideation and/or engaging in suicidal behaviour. Future research should also investigate the experiences and perceptions of these professionals, for example those working in primary care, maternity and midwifery services and private counsellors. The findings would provide insights into how professionals who work with all mothers (not just those who have reported mental health difficulties) identify those who have suicidal thoughts, encourage these mothers to talk about their thoughts and how they support these mothers.

## Conclusion

Determining what might contribute to or reduce suicidal ideation and behaviour in perinatal mothers is complex and nuanced. Our findings suggest that perinatal mental health professionals perceive the support around the mother, whether the pregnancy was planned and what the baby represents for the mother as important factors to consider when caring for a mother with mental health difficulties and responding to her risk of suicidal ideation and behaviour. Professional narratives underscored the importance of a tailored approach when discussing suicidal thoughts, plans and behaviours to encourage disclosure. Emphasising a mother’s unique importance to her own baby could be helpful when working to reduce suicidal ideation. Future research should focus on establishing if mechanisms linking self-efficacy and mother-infant bonding with suicidal outcomes exist.

## Data Availability

The datasets generated and analysed during the current study are not publicly available due to the sensitive nature of the interviews. However, they are available to bona fide researchers from the corresponding author on reasonable request.
